# Finding order within the disorder: a case study exploring the meaningfulness of delusions

**DOI:** 10.1192/bjb.2020.151

**Published:** 2022-04

**Authors:** Rosa Ritunnano, Clara Humpston, Matthew R. Broome

**Affiliations:** 1 Institute for Mental Health, University of Birmingham, UK; 2 Early Intervention in Psychosis Service, Coventry and Warwickshire NHS Partnership Trust, UK; 3 Early Intervention in Psychosis Service, Birmingham Women's and Children's NHS Foundation Trust, UK

**Keywords:** Delusion, phenomenology, psychopathology, psychosis, meaning

## Abstract

Can delusions, in the context of psychosis, enhance a person’s sense of meaningfulness? The case described here suggests that, in some circumstances, they can. This prompts further questions into the complexities of delusion as a *lived* phenomenon, with important implications for the clinical encounter. While assumptions of meaninglessness are often associated with concepts of ‘disorder’, ‘harm’ and ‘dysfunction’, we suggest that meaning can nonetheless be found within what is commonly taken to be incomprehensible or even meaningless. A phenomenological and value-based approach appears indispensable for clinicians facing the seemingly paradoxical coexistence of harmfulness and meaningfulness.

The prevailing clinical view of delusions in the context of psychosis, as reflected in the cognitive literature, supports the central proposition that delusions are the result of disturbances of reasoning and that they represent maladaptive appraisals about the world one inhabits. Such inferences are often taken to be grossly outside of the realm of psychological comprehension and, for the most part, inherently dysfunctional. In some cases, this can lead to prejudiced attributions of bizarreness, irrationality and meaninglessness. Very few empirical studies have investigated the relationship between delusions and meaningfulness. In addition, since the so-called ‘operational revolution’ in psychiatry, a tendency to focus on notions of disorder, disability and distress has often led to a reduced attention to issues of subjectivity, personhood and meaningfulness within the experience of illness. Through presenting this case study, we wish to bring these issues back to the fore.

## The case of Harry, the ‘happiest man in the world’

Mr Harry is a 33-year-old gentleman who has been complaining of being the target of a worldwide conspiracy for the past 5 years. He explains that one day, he was in his room and he was picking his nose. The cameras in his room recorded it and this was uploaded to the internet; now everyone in the world, especially those in the USA, are talking about it. People in the streets make signs like scratching their nose or straightening their hair, as an indication to him that they have seen his videos and know about him. He believes that there is a hierarchy of people who are taunting him, and this hierarchy goes up to the Illuminati and the Queen. He thinks that there are lizard people in charge of the Earth that have made people believe that the Earth is round, when in fact it is flat and ends at the Arctic and Antarctic (where there are ice cliffs beyond which we are not allowed to go). He believes that people are being told lies since the day they are born, and two- and three-dimensional imagery are used by the National Aeronautics and Space Administration to fool people into believing what they see is real, just like in the film ‘The Truman Show’. Harry thinks that scientists are trying to fool humanity by leading us away from religion. Satan is taking over the world and the Earth is moving at 6660 miles per hour.

During a consultation with one of the authors (R.R.), Harry explained how he knows all this: ‘My senses have shown me that nothing is real, there is nothing outside this world, just infinite space’. These experiences are unique to him and he feels very special as a result, as nobody else has access to the truth. He reported watching YouTube videos that have proven all his theories, and now fully understands what the world is really like. He is the happiest man in the world and states that every day is filled with novelty and excitement. Harry finds it surprising that people are blind to all this, and have nothing better to do in their lives than following him around, listening to his thoughts and controlling him. However, he has now learnt that he can reply back and influence their thoughts positively, which has made him feel more powerful than he has ever felt. Harry denies ever feeling threatened or anxious when he found out about the conspiracies, although he has not been able to work because of this outside interference. When asked further about the challenges of conducting a life under the control of others, Harry replied:

‘If I went out one day and I realised that people weren't expecting me to be there, it would be a real shock again …I would be … I don't know …?! I got so used to people expecting me to be there and lash out with them …I would feel alone again, which is what everyone else feels, like alone. So people are like a family for me, it's like a safety blanket, they make me feel so comfortable now …If I found out that they are not watching me and reading my mind, I would feel alone and crazy like everyone else. To feel like I have everyone following me around, whether it's negative or positive, that alone is a force of power … knowing that you can influence people's minds in the right way, I feel like Jesus (of course I'm not) but why not believe?’

## Clinical encounter

Harry was referred to Early Intervention in Psychosis services via his general practitioner, after revealing the unusual situation in which he found himself. He was prompted to seek help by his family, who were concerned about his mental health, although he did not believe he needed help. Rather, he thought that everyone else was deluded for not realising what was really happening. At the time of the first interview with the psychiatrist, Harry was described as a very pleasant, cooperative gentleman, and caring father. Upon examination, his content of thought was characterised by ever-increasing systems of conspiratorial views of the world and described as bizarre – including, for example, the idea that the Queen is a lizard in disguise. This was associated with a mild degree of formal thought disorder and decline in social and occupational functioning. There was no evidence of marked changes in mood, hyperactivity or other signs of elation. Harry described ‘voices’ talking to him and about him, although when asked the direct question of whether he was ‘hearing’ voices, he denied this. Risk to others was considered to be low, whereas a potential vulnerability to others was highlighted.

Given the presence of clear positive psychotic features and impairment in functioning, suggesting first-episode psychosis, Harry was informed about treatment options and antipsychotic medication was prescribed. At the following appointment, Harry reported non-adherence with his medication. He said that he had realised there was a microchip inside him that was being used by others to monitor him and read his mind. People were making signs at him, following him around, thus he needed to ‘deal with them’. He insisted that he was ‘completely in control’ and that those who were persecuting him were the ones needing help. While discussing medication, he became irritable and started accusing staff of considering him ‘crazy’. He was not amenable to persuasion or reasoning. To avoid escalation, the clinician had no option but to let him leave for his home.

Management options were discussed, and clinicians were divided between those who felt that Harry's symptoms should not be necessarily considered pathological, and those who felt that his presentation would fulfil the criteria for compulsory admission to hospital. This latter understanding was motivated by the clear evidence of mental disorder, a detected risk of him acting out on his beliefs that others were persecuting him, potential risk (but no evidence of this) to his children and failed attempts to treat him in the community. Initial diagnostic considerations made by the treating clinician were in keeping with a diagnosis of schizophrenia. However, this diagnosis was challenged by others, who suggested that his delusions were better explained by a delusional disorder or, more simply, were in keeping with the influence of cultural factors and were not pathological at all. Harry remained convinced throughout that his experiences were real, that he was the happiest man in the world and that clinicians had no good reason to ‘label’ him as mentally ill or recommend a compulsory admission. Unfortunately, the disagreement led to significant subjective distress reported by Harry, a breakdown in the therapeutic relationship, disengagement from the team and other adverse social consequences, including denial of access to his children for a period of time.

## Questions concerning current conceptions of delusions

This case is illustrative of more general, unresolved issues concerning current conceptions of delusions, which have a knock-on effect on the clinical encounter with deluded patients (irrespective of their diagnosis, but potentially more relevant in the case of schizophrenia). In particular:

- What exactly is pathological about delusions? This is briefly discussed in *Delusional complexities*.
- Are delusions the source of the problem or a response to the problem? This is addressed in *The phenomenological approach to delusion formation*.
- Can delusions have and give meaning? An overview of the small body of relevant literature is offered in the corresponding section. Our inquiry into meaning in this context takes a subjectivist naturalist perspective on the conception of ‘meaning’. Meaning refers here to the extent to which one's life is subjectively experienced as making sense, and as being motivated and directed by valued goals.

We temporarily leave diagnostic challenges aside and explore possibilities for a cross-disciplinary dialogue between philosophy and psychiatry concerning the nature and meaning of delusions, with direct relevance for clinical practice. Implications for the clinical encounter are discussed in the final section.

## Ethical considerations

The reported patient agreed to the publication of the case study and provided written consent. All steps were conducted in accordance with the regulations of Coventry and Warwickshire Partnership NHS Trust and the Declaration of Helsinki. Written approval for the publication of the report was obtained from the Research & Innovation Department, Coventry and Warwickshire Partnership NHS Trust.

## Delusional complexities

Delusions are core psychopathological features of severe mental illness. They are present in the vast majority of patients at first presentation to early intervention services across affective and non-affective diagnoses within the psychosis spectrum.^1^ They are often associated with great distress, depression and harm, representing a significant therapeutic challenge for clinicians.^2,3^ Despite extensive literature on the potential psychological, neurocognitive and phenomenological underpinnings of delusion formation,^4–6^ there is no consensus as to what causes delusions or why they are maintained despite their harmful consequences. Furthermore, cross-disciplinary attempts to define their puzzling nature remain inconclusive – perpetuating the philosophical debate between doxasticists (who regard delusions as beliefs) and non-doxasticists (who regard delusions as other than beliefs).

In psychiatric practice, given the absence of clear biological markers, the distinction between delusional and non-delusional ideas is not straightforward. Although meta-analyses of the available data corroborate a connection between reasoning biases and the occurrence of delusional ideas,^7^ they do not provide an explanation as to why delusions have the specific thematic content that they have, nor do they establish clear evidence for a causal relationship. Given the difficulties in defining what kind of phenomena delusions are, and in identifying the aetiological factors involved in their formation and maintenance, the clinical examination and study of delusions continue to focus on their (apparently more reliable) doxastic features. Such features predominantly consist of negative epistemic attributes such as falsehood/incorrectness, fixity/resistance to counterargument and counterevidence, and implausibility of content.^8^ For example, the DSM-5 defines delusion as:

‘A false belief based on incorrect inference about external reality that is firmly held despite what almost everyone else believes and despite what constitutes incontrovertible and obvious proof or evidence to the contrary. The belief is not ordinarily accepted by other members of the person's culture or subculture (i.e. it is not an article of religious faith). When a false belief involves a value judgment, it is regarded as a delusion only when the judgment is so extreme as to defy credibility. Delusional conviction can sometimes be inferred from an overvalued idea (in which case the individual has an unreasonable belief or idea but does not hold it as firmly as is the case with a delusion)’ (p. 819).^9^

However, as philosophers have already made clear, overreliance on these criteria is often not a successful strategy when trying to distinguish pathological beliefs from everyday irrational beliefs.^10^ For instance, prejudiced, superstitious or self-enhancing beliefs are all often ill-grounded and impervious to counterargument, yet they do not warrant a psychiatric diagnosis or compulsory treatment. In the case of Harry, these criteria evidently fell short and clinicians immediately noticed how similar Harry's belief were to those held by ‘flat earthers’ and other fringe communities with heavy influence across social media. What then makes such beliefs different from those of patients affected by schizophrenia? When do beliefs become a symptom of mental disorder?

Focusing on the psychological and sociological features (such as distress, harm and dysfunction) associated with certain unusual convictions might be a better way forward for clinicians. However, this pragmatic approach, relying on criteria of clinical utility, hides other significant and ethically loaded challenges.^11^ For instance, how do we equitably decide on the threshold of harm or potential harm that deserves a psychiatric diagnosis and/or warrants treatment against someone's will? Such a decision will necessarily involve a value judgement on the part of the clinician not only about what might be harmful to another person in relation to their behaviour, but also in relation to their own feelings (e.g. levels of distress), sociocultural background, previous life circumstances and future goals. Disregarding the value-laden context that shapes the lived experience of delusional phenomena might increase patients’ vulnerability to suffering epistemic injustice.^12^ Harry for example, appeared to be adequately fulfilling his parental role and repeatedly denied feeling distressed, anxious, worried or depressed. A battery of psychological tests showed no clinically relevant anxiety or depression; rather, they revealed surprisingly high levels of meaning in life (see discussion below). On the other hand, his level of social and occupational functioning is moderately low. Harry is unable to maintain a stable occupation and what seems to be giving a special significance to his experience (i.e. the fact that he has special access to the truth) is effectively making him an outcast from society.

Clinicians therefore seem to be faced with a case of meaningful dysfunction. The person's beliefs seem to impose a limitation on their objective ability to keep consistent employment (social dysfunction). Concurrently, they also seem to enhance the person's sense of agency and belonging, and no distress is reported with regards to either the beliefs or the ensuing impairment. Does such a condition deserve clinical attention? Does it require pharmacological treatment? Despite being grounded within a delusional experience, could such feelings play a protective role against depression and anxiety?

It is clear that, although the concept of delusions as ‘false beliefs’ is commonly taken for granted within mainstream psychiatry, their complex nature remains difficult to grasp. As a result, the threshold for pathology or dysfunction continues to be set on pragmatic grounds relying on criteria of severity and degree of distress/functional impairment. However, in certain cases, it seems that the clinical utility of pragmatic criteria is limited by a clash with the framework of values of the individual patient. This begs the key question of what constitutes a meaningful or functional life, and leads us further into the relationship between facts and values in psychiatry.^11^

Although many of these questions remain open and in need of further philosophical investigation, an important response in the past 20 years has been the renewed interest in phenomenological approaches to psychopathology. This has been accompanied by a revival of the legacy of Karl Jaspers and other classical authors, such as Minkowski, Bleuler, Conrad, Blankenburg, Mayer-Gross and J.S. Strauss, among others.^13–17^ The phenomenological approach argues that, particularly in the case of schizophrenia, there is a qualitative difference between ‘true’ delusions and delusion-like ideas, and that a more precise and in-depth characterisation of changes in the experience of self and lived world is needed if we aim to distinguish non-disordered analogues from clinically relevant forms of psychopathology.

## The phenomenological approach to delusion formation

Various phenomenologically informed authors have challenged the view that delusions are beliefs (see Table 1 for some excerpts from the contemporary phenomenological literature). In contrast with the doxastic (i.e. belief-based) position, phenomenologists have understood delusions to be either something of a completely different nature from beliefs (this is the ‘non-doxastic’ view), or they have suggested that this discussion is beside the point as it is failing to engage with what is most fundamental to delusion.^18^ Jaspers himself wrote: ‘To say simply that a delusion is a mistaken idea which is firmly held by the patient and which cannot be corrected gives only a superficial and incorrect answer to the problem. Definition will not dispose of the matter’ (p. 93).^19^

**Table 1:** Conceptions of delusions from a phenomenological perspective

‘For the phenomenologist, delusion is typically understood not as an individual belief […] but as a mutation of the ontological framework of experience itself.’ (p. 633)^20^
‘It follows that delusions, at least in this scenario, are not simply anomalous beliefs or perceptions. […] They have a type of intentionality that differs from mundane experiences of believing, remembering, imagining or perceiving.’ (p. 153)^21^
‘One might indeed argue that the so-called ‘delusional beliefs’ are not beliefs in the epistemic sense at all, for they lack the basis of a shared intentional relation to the world.’ (p. 25)^22^
‘Schizophrenic delusions typically reflect a fundamentally altered existential-ontological structure of subjectivity.’ (p. 173)^23^
‘When a subject enters into a delusional state, he or she is entering into an alternative reality. [*…*] one can enter into a delusional reality just as one can enter into a dream reality, or a fictional reality, or a virtual reality.’ (pp. 255–6)^24^

Following Jaspers, much phenomenological research has drawn attention to the subtle and all-enveloping changes that are often described by patients with delusions during the ‘prodromal’ or ‘pre-delusional’ stages. Jaspers refers to this experience as ‘delusional mood’ or ‘delusional atmosphere’, and describes it as follows:

‘Patients feel uncanny and that there is something suspicious afoot. Everything gets a new meaning. The environment is somehow different—not to a gross degree—perception is unaltered in itself but there is some change which envelops everything with a subtle, pervasive and strangely uncertain light. A living-room which formerly was felt as neutral or friendly now becomes dominated by some indefinable atmosphere. Something seems in the air which the patient cannot account for, a distrustful, uncomfortable, uncanny tension invades him’ (p. 98).^19^

In Jasper's view, the subsequent emergence of a specific belief content can only be understood in the context of a ‘transformation in our total awareness of reality’.^19^ Such fundamental transformation can, in some cases, give rise to what he calls ‘delusion proper’ or ‘primary delusions’ to distinguish them from ‘delusion-like ideas’. Although the latter kind of delusional beliefs can be understood as an excess or lack of certain known emotional states or responses (such as fear, melancholy, suspiciousness, anxiety and wonder), the former kind of delusions remain largely incomprehensible in the face of empathic or common-sense attempts to grasp their meanings.

Just as Harry mentioned the film ‘The Truman Show’ to aptly communicate his puzzling experience of infinite space, many patients talk about living in a ‘real simulation’ or a ‘fake reality’ to convey the sense of unreality that surrounds them. In these moments, they often describe changes in their subjective experience of the lived world, including the dimensions of time, space, objects, atmospheres and other persons.^25^ For example, time or movements might be experienced as accelerated or slowed down, objects may appear two-dimensional as if they were artificially projected on the backdrop of a theatrical scenery, and other people may look like mannikins, puppets or robots wearing a mask.^26^ This is similar to what Renee describes as an all-embracing atmosphere of unreality in her memoir:

‘Objects are stage trappings, placed here and there, geometric cubes without meaning. People turn weirdly about, they make gestures, movements without sense; […]. And I - I am lost in it, isolated, cold, stripped purposeless under the light. A wall of brass separates me from everybody and everything. In the midst of desolation, in indescribable distress, in absolute solitude, I am terrifyingly alone; no one comes to help me. This was it; this was madness […] Madness was finding oneself permanently in an all embracing Unreality’ (p. 33, abridged).^27^

Although this can be perceived in some cases as an exciting and illuminating experience (such as in Harry's case), most often the delusional atmosphere is fraught with dread, anxiety and a sense of uncertainty. Patients often describe an increasing tension coupled with an unbearable sense of impending doom.

In his seminal work, the German psychiatrist Klaus Conrad calls this initial phase ‘trema’ (stage fright) – emphasising the suspenseful and expectational character of the experience.^28^ Even Harry reported that it all came as a shock for him, calling into questions everything he knew about the world since the day he was born. This state of perplexity seems to trigger an urgent quest for meaning, as highlighted in many first-person reports and clinical accounts.^29^ The delusion then provides the long-sought meaning that dissipates anxiety, perplexity and confusion. In this moment, which Conrad calls the ‘apophany’ or ‘aha experience’, the person promptly makes sense of what was previously only alluded to. This new (delusional) meaning alleviates the unbearable sense of dread previously felt. The soothing effect provided by the experience of finding ‘a fixed point’ to cling on is described well by Jaspers:

‘This general delusional atmosphere with its vagueness of content must be unbearable. Patients obviously suffer terribly under it and to reach some definite idea at last is like being relieved from some enormous burden […] the achievement of this brings strength and comfort, and it is brought about only by forming an idea, as happens with health people in analogous circumstances’ (p. 98, abridged).^19^

Framed in this way, the newly developed delusional framework can be understood as establishing a new ‘order’ within the ‘disorder’, one which can alleviate negative feelings of anxiety or induce intense feelings of wonder. This allows the person to re-establish a pragmatic connection with the world, although this can come at great expense because of the difficult integration between the shared sociocultural world and the delusional reality. Rather than being the source of the problem, the emerging delusional narrative (i.e. what we currently identify as belief) may be better interpreted as a secondary response to anomalous experiences which call into question our most fundamental assumptions about ourselves, the world and the meaning of life.

## Can delusions have and give meaning?

After a period of disengagement with services, Harry agreed to continue working with the team, although he refused to interact with staff initially involved in his care. Because of the research interests of one of the clinicians (R.R.), Harry was invited to talk about his experiences, and he happily completed a small battery of self-administered psychological tests that measure depression (Calgary Depression Scale for Schizophrenia),^30^ anxiety (Generalized Anxiety Disorder seven-item scale)^31^ and meaning in life (the Purpose-in-Life Test (PILT), the Life Regard Index (LRI) and the Multidimensional Existential Meaning Scale).^32–34^ These assessments revealed high scores across three measures of meaning in life (indicative of a strong sense of coherence (SOC), purpose and significance), and low scores on the depression and anxiety scales, suggestive of absent levels of depressive or anxious features (see Table 2).

**Table 2:** Self-administered measures of depression, anxiety and meaning in life conducted in the case study

Measure	Total score	Details of measures
Calgary Depression Scale for Schizophrenia	3	≥6 is commonly used to identify clinically significant depressive symptoms
Generalized Anxiety Disorder seven-item scale	0	Scores of 5, 10 and 15 are taken as the cut-off points for mild, moderate and severe anxiety, respectively
Purpose-in-Life Test	96	Range 20 (low purpose) to 100 (high purpose)
Life Regard Index	68	Range 14 (low life regard) to 70 (high life regard)
Multidimensional Existential Meaning Scale	99	Range 15 (low existential meaning) to 105 (high existential meaning)

There is no doubt that Harry's experiences have brought about a significant change in the way in which Harry sees himself and the world around him, albeit one that others cannot recognise. As we can gather from his account, it all came as a shock, a powerful revelation of what life is really like. Whether this change is one that can be understood by others as ‘having meaning’ (i.e. making sense) and ‘giving meaning’ (i.e. contributing to a sense of purpose and significance) is a far more complex issue, but one worthy of further investigation and one that carries significant implications for the clinical encounter. From Harry's perspective, this new order seems to provide a coherent explanation for his experiences, while also enhancing his sense of direction in life and enthusiasm regarding the future.^35^ There is, however, a remarkably small amount of empirical research that has examined such issues, which we briefly review below.

In a study by Roberts,^36^ a group of patients with chronic schizophrenia displaying elaborated delusional systems was administered the PILT and the LRI. The author compared the scores obtained by actively delusional patients with chronic schizophrenia with a matched sample of other chronic patients, who were previously deluded but were now in remission. Psychiatric rehabilitation nurses and Anglican ordinands were also included as non-clinical comparison groups. Results showed that patients with elaborated delusions had a very high level of perceived purpose and meaning in life (and low level of depression and suicidal ideation), and PILT/LRI scores were significantly higher than those found in patients with chronic schizophrenia in remission. The group in remission felt both more depressed and found their lives less meaningful than those with active delusions. Scores in the actively deluded group were also similar to those found in the Anglican ordinands comparison group and higher than those found in the nursing group. Another study^37^ investigated the relationship between the SOC and delusional experiences in individuals with schizophrenia, using self-report scales for delusions, SOC, depression and expressed emotion. SOC among participants experiencing acute delusion was found to be similar to the average scores found in the general population, but a reduction in SOC was found in the remission period, suggesting decreased well-being among those with reduced delusional intensity. These findings led Bergstein et al^37^ to speculate about the subjective meaning-enhancing effect of delusional systems, and the potential negative consequences associated with the undermining of the acquired (delusional) background of meaning.

More recently, Isham et al^2^ conducted a qualitative analysis of the narratives of 15 patients with past or present experiences of grandiose delusions. Although suggesting that serious harm (including social, physical, sexual, emotional and occupational) was occurring to people as a result of the delusions, the narratives examined contained first-person descriptions of the grandiose beliefs as highly meaningful: a meaning-making theme was generated through the analysis, where the delusion seemed to ‘provide a sense of purpose, belonging, or self-identity, or to help make sense of unusual or difficult events.’ A highly prevalent theme was related to social meanings (i.e. being useful to and a significant part of society), whereby participants felt ‘part of a team’, respected by others or involved in intimate relationships. Similarly, in their qualitative in-depth analysis of four cases, Gunn and Larkin^38^ describe the development of delusions as an ‘inevitable consequence of a radical alteration in lived experience’. Focusing on what was important to the participants and grounding their interpretation in the data by using interpretative phenomenological analysis, they highlight how all their participants had experienced some perceptual, affective and emotional anomalies demanding explanatory and sense-making attempts. Although these attempts turn out to be delusional, they nonetheless seem to provide a fitting explanation for the anomalous experiences, as well as potential psychological benefits in terms of enhanced self-efficacy and meaningfulness.

## Implications for the clinical encounter

Harry's case highlights the complexities intrinsic to the concept and nature of delusions, which are commonly taken for granted within mainstream psychiatry practice. By appealing exclusively to surface epistemic features, Harry's delusions might appear outwardly almost indistinguishable from fringe conspiracy beliefs. In both cases, they are ill-grounded and we have reasonable contradictory evidence regarding their veracity. Harry (just like many conspiracy theory believers) is not be amenable to changing his mind about the fact that he is constantly monitored, that the Queen is a reptile in disguise and that the Earth is flat, among other more systematised convictions. His beliefs are certainly fixed and impervious to counterargument. Do these features make them pathological? By appealing to a pragmatic criterion of harmful dysfunction, we could agree on the fact that Harry's social and occupational functioning is impaired and therefore adequate interventions should be sought – aiming to ameliorate such undesirable state. However, Harry is telling us that he is the happiest man in the world. He reports finding a highly significant meaning for leading his life, something that gives him coherence and purpose. Value judgements necessarily come into play at this point, raising broader and more challenging questions about what makes a good life and where the threshold should be set for something meaningful to become harmful. Although we may not have a clear answer to these questions, we should at least attempt to investigate what the world feels like for Harry. Such phenomenological endeavour might not only open up a space for dialogue, but can also advance our understanding of the nature and constitution of delusional phenomena. Just like the three blind men who came to different conclusions as to the nature of an elephant, looking only at the ‘belief’ side of delusions might limit our understanding of what makes the delusional experience possible in the first place. This may further aid our attempts to define what makes delusions pathological or when they should be considered part of a disorder.

Taking into account the subjective changes to the sense of self and world often affecting people with delusions can improve our empathic understanding of delusional phenomena; that is, as arising in the context of a more global transformation of the sense of reality and familiarity. Within the clinical encounter, delusions can be at the same time harmful (e.g. causing a dysfunction of some kind) and meaningful. They can have meaning (i.e. make sense) in relation to uncanny changes in the lived world, and they can give meaning (i.e. purpose/significance) in the context of the person's unique life story and framework of values. When a clash of realities creates an impasse within the clinical encounter, clinicians should investigate the presence of anomalous and potentially distressing changes in the subjective experience of the lived world. Clinicians should also acknowledge the relentless sense of perplexity often arising from these experiences, which might trigger a search for explanations and a quest into the meaning of existence. Although empirical research into these issues is at its infancy, the potential role of feelings of meaningfulness in the maintenance of delusions (and their potential subsiding after remission) should be considered throughout the engagement and recovery processes. Further interdisciplinary research is needed to address the question of what constitutes meaningfulness and to explore its relationship with mental illness.

## About the authors

**Rosa Ritunnano**, MD, is a consultant psychiatrist with the Early Intervention in Psychosis Service at Coventry and Warwickshire NHS Partnership Trust, UK, and a PhD candidate at the Institute for Mental Health, University of Birmingham, UK. **Clara Humpston**, PhD, is a research fellow in youth mental health methodology at the Institute for Mental Health, University of Birmingham, UK. **Matthew R. Broome**, PhD, FRCPsych, is Director of the Institute for Mental Health, University of Birmingham, UK, and an honorary consultant psychiatrist with the Early Intervention in Psychosis Service at Birmingham Women's and Children's NHS Foundation Trust, UK.
